# Total scattering measurements at the Australian Synchrotron Powder Diffraction beamline: capabilities and limitations

**DOI:** 10.1107/S1600577522011614

**Published:** 2023-01-13

**Authors:** Anita M. D’Angelo, Helen E. A. Brand, Valerie D. Mitchell, Jessica L. Hamilton, Daniel Oldfield, Amelia C. Y. Liu, Qinfen Gu

**Affiliations:** a Australian Synchrotron, Australian Nuclear Science and Technology Organisation (ANSTO), 800 Blackburn Road, Clayton, Victoria 3168, Australia; b Australian Nuclear Science and Technology Organisation (ANSTO), New Illawarra Road, Lucas Heights, New South Wales 2234, Australia; c School of Physics and Astronomy, Monash University, Wellington Road, Clayton, Victoria 3800, Australia; Paul Scherrer Institut, Switzerland

**Keywords:** pair distribution function, powder X-ray diffraction, Mythen-II detector, total scattering

## Abstract

This study describes the capabilities and limitations of carrying out total scattering experiments on the Powder Diffraction (PD) beamline at the Australian Synchrotron, ANSTO. A maximum instrument momentum transfer of 19 Å^−1^ can be achieved if the data are collected at 21 keV. The results detail how the pair distribution function (PDF) is affected by *Q*_max_, absorption and counting time duration at the PD beamline, and refined structural parameters exemplify how the PDF is affected by these parameters. There are considerations when performing total scattering experiments at the PD beamline, including (1) samples need to be stable during data collection, (2) highly absorbing samples with a μ*R* > 1 always require dilution and (3) only correlation length differences >0.35 Å may be resolved. A case study comparing the PDF atom–atom correlation lengths with EXAFS-derived radial distances of Ni and Pt nanocrystals is also presented, which shows good agreement between the two techniques. The results here can be used as a guide for researchers considering total scattering experiments at the PD beamline or similarly setup beamlines.

## Abstract 2

Total scattering data can be obtained on the PD beamline although there are constraints to the capabilities offered including longer acquisition times and necessary dilution of highly absorbing samples. This work details the considerations needed to successfully carry out total scattering studies on this bending-magnet beamline, which has not been specifically designed for total scattering experiments.

## 1. Introduction

In a typical powder diffraction experiment from a crystalline specimen, where only the Bragg reflections are studied, only information on the average long-range order of atomic structures is available. However, further insight into the functional properties may be obtained through understanding the local structure by analysing both the sharp Bragg and the diffuse scattering from disorder via total scattering analysis. Both Bragg and diffuse scattering are considered in total scattering experiments and the pair distribution function (PDF) is obtained through Fourier transform of the total scattering data.

A PDF shows the probability of finding an atom at distance *r* from a specified atom. The technique has applications for nanoparticles, disordered materials, non-periodic materials such as amorphous glasses, as well as systems where the local structure is different to the average structure. PDF measurements can provide information on a short- and medium-length scale and are complementary to other techniques such as extended X-ray absorption fine structure (EXAFS). EXAFS provides local information up to the first three atomic shells or ∼6 Å as the signal attenuates dramatically when the distance between the neighbouring and absorbing atom increases. In contrast, a PDF can provide information beyond 1000 Å depending on the instrument resolution in reciprocal space. EXAFS is used to target the local structure of a specific element whereas PDF provides local structure information from all atom pairs. However, this means pairs close to each other can be hard to resolve using PDF. Structural details that can be examined include (1) the direct atom pair separation length from peak positions, (2) the degree of disorder of the atom pair from the peak width, (3) static and dynamic disorder, (4) the phases present in mixed-element systems using differential PDFs, (5) details about specific atom–atom correlations via partial PDFs and (6) coordination number from the integrated peak intensity if the data are on an absolute scale (Takeshi & Billinge, 2012 ▸).

High counting statistics are needed as both the Bragg and the diffuse scattering contribute to the PDF. The atomic X-ray form factor decreases with increasing *Q*, which contributes to a decrease in Bragg peak intensity and non-uniform counting statistics. The *S*(*Q*) is obtained by subtracting self-scattering and normalizing the coherent scattering intensity per atom *I*_coh_(*Q*), according to equation (1) where *Q* is the magnitude of the scattering vector, *c_i_* is the atomic concentration and *f_i_* is the X-ray form factor for atom type *i* (Farrow & Billinge, 2009 ▸; Billinge & Farrow, 2013 ▸). The reduced structure function *F*(*Q*) is determined from *S*(*Q*). A Fourier transform is applied to the normalized total scattering data to generate the reduced PDF *G*(*r*), where ρ_0_ is the number density, *g*(*r*) is the PDF, *Q* is the magnitude of the wavevector transfer, *S*(*Q*) is the structure function and *r* is the distance between two atoms in the sample [equation (2)] (Farrow & Billinge, 2009 ▸). Note there are many different total scattering correlation functions, of which their definition also varies (Keen, 2001 ▸),

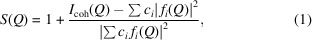




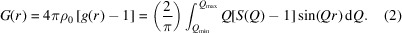

High-energy X-rays are generally used in PDF experiments to increase the momentum transfer and real space resolution. For example, experiments have been carried out using a resolution of 0.126 Å for 28-ID-1 at the National Synchrotron Light Source II, and 0.0898 Å for the General Materials Diffractometer (GEM) at ISIS Neutron Source of Rutherford Appleton Laboratory (Frandsen *et al.*, 2019 ▸). A disadvantage of lower real space resolution is that correlation lengths that are similar cannot be resolved. This is because the limits of the *G*(*r*) function are determined by the *Q*-range. As an example, the Zn—Se and Zn—Te bonds in ZnSe_0.5_Te_0.5_ cannot be distinguished at *Q*_max_ = 17 Å^−1^ as the bond length difference is 0.14 Å, whereas they are clearly visible at *Q*_max_ = 40 Å^−1^ (Proffen *et al.*, 2003 ▸). A high *Q*-range can typically be achieved in two ways: (1) increasing the beam energy if using a flat-panel 2D detector, or (2) increasing the 2θ angle using a point or 1D detector if the maximum energy is limited. PDFs can also be generated using neutron or electron sources. PDFs generated from electrons (ePDF) allow for smaller samples to be analysed and data with higher spatial resolution can be obtained (McBride & Cockayne, 2003 ▸, 2007 ▸). Neutron PDF experiments are commonly carried out at spallation sources including ORNL, ISIS, J-PARC and the European Spallation Source. Reactors such as D4 at Institut Laue-Langevin can also generate short-wavelength neutrons (*i.e.* 0.35 Å and covering 1.5 to 140° 2θ) with a hot source that makes them suitable for total scattering studies (Fischer *et al.*, 2002 ▸).

Synchrotron hard X-ray sources are generally preferred over laboratory X-ray sources due to the higher energy and higher flux available. The energy used on dedicated PDF beamlines is typically >60 keV, *i.e.* 76.6 keV at I15-1 at Diamond Light Source, 86.7 keV at 11-ID-B at the Advanced Photon Source (APS) and up to 117 keV at 28-ID-1 at Brookhaven National Laboratory. However, reasonable values of *Q*_max_ can be achieved using lower-energy X-rays such as those generated in the laboratory using Mo *K*α (17.4 keV) (Rantanen *et al.*, 2019 ▸; Galliez *et al.*, 2014 ▸) and Ag *K*α (22.1 keV) (Wu *et al.*, 2018 ▸; Bueken *et al.*, 2017 ▸) radiation. Dykhne *et al.* (2011 ▸) detailed a comparison of PDF data generated from laboratory X-ray sources for pharmaceutical applications. Furthermore, PDF studies have also been carried out on lower-energy beamlines such as DanMAX at MAX IV with a maximum energy of 35 keV. PDF studies at the X-ray Diffraction and Spectroscopy (XDS) beamline at the Brazilian Synchrotron Light Laboratory (LNLS) have been carried out at 20 keV with *Q*_max_ = 20 Å^−1^ using a Mythen-II detector by collecting between 1° and 165° 2θ, and the results were compared with PDF studies carried out at the 11-ID-B PDF beamline at the APS (Saleta *et al.*, 2017 ▸). In their work the authors reported that XDS can obtain high-quality PDF data and is preferential to study *r* regions >30 Å in comparison with the APS. This was attributed to a smaller dampening factor calculated from XDS compared with APS. Despite this, studies carried out at lower energies may also only report qualitative results due to the limited resolution achieved.

The Australian Synchrotron is a third-generation light source that operates at 3 GeV and 200 mA during user operations. The PD beamline is optimized to carry out *in situ* studies in a broad range of fields including energy, condensed matter science, materials engineering and environmental science. It is located on a bending magnet with a magnetic field of 1.3 T and a critical energy of 7.78 keV. After several years of user operations, the PD beamline optics were upgraded and simplified to better meet the needs of the user community. In the original beamline design the double-crystal monochromator (DCM) had both a flat Si(111) and an Si(311) flat/sagittally bent crystal pair. The mirrors were also designed with multiple stripes for optimal reflectivity over the full energy range of the beamline (4–30 keV). The recent upgrade removed the Si(311) crystal pair and redesigned the crystal mounting for added stability as well as upgrading the mirrors to add horizontal focusing. The vertical collimating mirror (VCM) and vertically focusing mirror (VFM) were replaced with a single-stripe Rh-coated flat and toroid mirror that provided additional flux through the ability to horizontally focus the beam to ∼0.8 mm × 0.6 mm in the first endstation. The useable range of energies on the beamline is now 8–22 keV as the reflectivity of the Rh mirror coating drops off substantially above 22 keV.

The PD beamline consistently receives requests to carry out total scattering experiments for various materials including battery electrodes, piezoelectrics and coordination framework materials. Early in user operations, studies were carried out by Haverkamp & Wallwork (2009 ▸) to assess the feasibly of PDF measurements at the PD beamline using the Mythen-II detector at 21 keV. In this work the need for a wide *Q*-range, fluorescence elimination and removal of the detector gaps between the detector modules (as per standard beamline procedure) were highlighted. The Mythen-II can achieve *Q*_max_ = 40 Å^−1^ if data are measured over 155° 2θ at 40 keV (Bergamaschi *et al.*, 2010 ▸). However, it is important to note that the Mythen-II detector efficiency at 5–10 keV is 85% and decreases to ∼25% at 20 keV for a 300 µm silicon wafer thickness that is used at the PD beamline. The efficiency could be improved by increasing the thickness of the silicon wafer. The Mythen-II has been used in PDF studies at the Materials Science Beamline (Swiss Light Source) with *Q*_max_ = 23 Å^−1^ (Černý *et al.*, 2009 ▸) and *Q*_max_ = 28 Å^−1^ (Saha *et al.*, 2021 ▸).

These recent beamline upgrades have prompted us to provide an update of the current PDF capabilities at the PD beamline. The aim of this study was to benchmark total scattering measurements carried out at the beamline. It demonstrates what can be offered to our user community through exploration of variations in *Q*_max_, absorption and counting time. A case study comparing the PDF atom–atom correlation lengths with EXAFS-derived radial distances is also presented.

## 2. Experimental

### 2.1. Materials

Ni powder (99.8%), Cu powder (10 µm spheroidal, 99%) and SiO_2_ nanopowder (10–20 nm, 99.5%) were purchased from Sigma–Aldrich. Ni, NiPt (1:1), dealloyed NiPt (1:1) and Pt metal nanoparticles (20 wt.%) supported on carbon were synthesized via the methods described by Wang *et al.* (2018 ▸).

### 2.2. Powder diffraction

Diffraction data were collected at the PD beamline at the Australian Synchrotron, ANSTO, using a Mythen-II detector over 124° 2θ using four detector positions (Bergamaschi *et al.*, 2010 ▸). Samples were packed into 0.3 mm borosilicate capillaries and rotated during data collection. The Mythen-II at the PD beamline has 16 modules that cover 80° 2θ and each module has an angular range of 4.83° with a physical separations of 0.17°. Patterns were obtained at starting angles of 2° 2θ (position 1), 2.5° 2θ (position 2), 51.5° 2θ (position 3) and 52° 2θ (position 4). The in-house-developed program *PDViPeR* was used to normalize and splice the four patterns and remove the gaps in the data. The wavelength, zero error and instrument contribution to the peak profile were determined using the line position and line shape standard NIST LaB_6_ 660b. The refined wavelength was 0.58907 (1) Å for the nanoparticle and Cu sample collections, and 0.58926 (1) Å for the Ni powder.

The instrument momentum transfer achievable by measuring 124° 2θ was 19 Å^−1^. An empty capillary was used as the background for the Ni and Cu powder. An SiO_2_-filled capillary was used for the Cu mixed with SiO_2_ samples. A capillary filled with carbon was used as the background for the metal nanoparticles so a PDF of the metal nanocrystal without a carbon contribution could be extracted. *xPDFsuite* was used to generate the *S*(*Q*), *F*(*Q*) and *G*(*r*) functions (Yang *et al.*, 2014 ▸) using *Q*_max_ = 18 Å^−1^. This software uses an *ad hoc* approach to correct and normalize the measured data before transformation to the PDF (Juhás *et al.*, 2013 ▸). The background scale was optimized to ensure that *S*(*Q*) oscillates around 1. *Q*_min_ = 0.5 Å^−1^, *r*_min_ = 0.01 and *r*_step_ = 0.01 were used for all data. *Q*_broad_ = 0.0017 (1) and *Q*_damp_ = 0.0035 (1) were determined from refining a NIST Si 640d standard in a 0.3 mm capillary between 1 Å and 2000 Å in *TOPAS* (version 6; Coelho, 2018*a* ▸,*b* ▸). Rietveld refinement and modelling of PDF patterns were carried out in *TOPAS* (version 6), and PDFs for NiPt, dealloyed NiPt and Pt were modelled up to 15 Å using a numerical spherical shape function (Usher *et al.*, 2018 ▸).

### 2.3. Transmission electron microscopy

Transmission electron microscopy (TEM) images for Ni were obtained on a JEOL 2200FS TEM at 200 kV. Images for the NiPt, dealloyed NitPt and Pt nanoparticles were obtained using a double-corrected FEI Titan 3 FEGTEM operated at 300 kV in scanning transmission electron microscopy (STEM) mode with a probe convergence semi-angle of 15 mrad. Bright-field (BF) and high-angle annular dark-field (HAADF) images were collected. Samples were prepared by adding a small quantity of the powder to ethanol and dropping 3 µl of the solution onto a Cu holey carbon Quantifoil grid then allowing to dry. Particle counting was carried out using *ImageJ* 1.53k via watershed image processing of the HAADF images. The average diameter of the nanoparticles was determined by fitting a Gaussian distribution to a scatter plot of the binned counts plotted as a function of particle size.

### 2.4. X-ray absorption spectroscopy

Samples were measured using X-ray absorption spectroscopy (XAS) at the XAS beamline at the Australian Synchrotron, ANSTO. XAS spectra were recorded at the platinum *L*_3_- and nickel *K*-absorption edges in fluorescence mode at 4 K in a helium atmosphere using a 100-element solid-state HP-Ge detector (Canberra/Mirion, France). The excitation energy was selected using an Si(111) DCM, which was calibrated at the Pt *L*_3_- and Ni *K*-absorption edges using inline metal foils (the first maximum of the first derivative was at 11562.76 eV and 8331.49 eV for Pt and Ni, respectively).

The samples were diluted to 1000 p.p.m. in cellulose and pressed into 7 mm pellets which were then loaded into Perspex holders and sealed with Kapton tape. The corresponding reference foils for Pt and Ni were analysed simultaneously with the appropriate samples. X-ray absorption fine structure (XAFS) scans were performed with a count time of 2 s for each energy step in the pre-edge and X-ray absorption near-edge spectroscopy (XANES) region, with 10 eV steps in the pre-edge region of the XAS spectra, and 0.25 eV steps in the XANES region. In the EXAFS region, spectra were collected in steps of 0.035*k* to a maximum of 20*k*, with count time increasing linearly from 2 s up to 10 s at the end of the EXAFS range. Data were pre-processed using the in-house program *Sakura* (Kappen *et al.*, 2015 ▸) and the *Demeter* package for normalization and EXAFS fitting (Ravel & Newville, 2005 ▸). The *R*-factors of the EXAFS fits obtained and presented in Section 3.5.2 were 0.005 and 0.004 for Ni and Pt, respectively.

## 3. Results and discussion

### 3.1. Instrument characterization

The effect of the instrumental resolution on the PDF was investigated using NIST Si 640d with a certified lattice parameter of 5.43123 (8) Å. Peaks dampen at high *G*(*r*) owing to changes in the effective angular resolution (Δ*Q*/*Q*) across *Q* in reciprocal space. The amplitude of the PDF peaks will dampen less at high *r* for high reciprocal space resolution instruments such as the PD beamline, which is optimized for high-resolution studies with an energy resolution of Δ*E*/*E* = 7.4 × 10^−4^ at 15 keV. The Mythen-II detector also provides an intrinsic angular resolution up to 0.004° with a 50 µm strip pitch (Haverkamp & Wallwork, 2009 ▸; Gozzo *et al.*, 2010 ▸). The intrinsic angular resolution is greater for the PD beamline compared with those beamlines that are optimized for PDF studies because of the beamline optics and the use of the Mythen-II instead of a 2D detector,

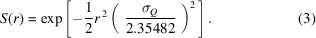

The Gaussian dampening envelope was determined by equation (3), where S(*r*) is the *r*-dependent scale factor, σ_Q_ is the width of the Gaussian determined by fitting the PDF (*Q*_damp_) to 2000 Å and *r* is the distance between two atoms in the sample. Fig. S2 of the supporting information shows the *r*-dependent scale factor up to 3000 Å. The degree of dampening is due to the *Q* resolution of the instrument and is advantageous when information of the medium-range atomic structure is required. For example, through modelling the crystalline contribution in the 40–60 Å region, correlations from nano-crystalline components up to 40 Å were extracted from cement data measured on the 11-ID-B beamline at the APS (White *et al.*, 2015 ▸). Work by Saleta *et al.* (2017 ▸) compared PDF data obtained on a Mythen detector at XDS at the LNLS with data obtained on 11-ID-B at the APS and showed that the PDF peaks attenuate quicker in the APS data. The LNLS PDF data were considered higher quality at higher *r* whereas the APS data were considered higher quality at lower *r*. The main advantage of the total scattering setup at the PD beamline is the lower dampening compared with typical high-energy PDF beamlines.

To investigate the effect of sample volume on the effective angular resolution in both real and reciprocal space, NIST Si 640d data were collected in both a 0.3 mm and a 1 mm capillary. Individual peaks were fitted with a pseudo–Voight and the full width at half-maximum (FWHM) determined. As expected, Fig. S3(*a*) shows that the FWHM increases as 2θ increases and the FWHM is larger for a 1 mm capillary compared with a 0.3 mm capillary. The FWHM of the Si(111) reflection was 0.0072 (1)° measured in a 0.3 mm capillary and 0.0142 (1)° in a 1 mm capillary. The effective angular resolution is lower for a larger sample volume as scattering extends over a greater angular range. The broadening contribution from a 0.3 mm capillary can span 0.006° versus 0.019° for a 1 mm capillary at a 762 mm sample-to-detector distance (Bergamaschi *et al.*, 2010 ▸; Gozzo *et al.*, 2010 ▸). Work by Scarlett *et al.* (2011 ▸) has also shown sample displacement artefacts in the diffraction data of minerals nucleating on the walls of 1 mm capillaries as the peaks shift from their ideal positions to higher and lower angles. These authors went on to formalize this observation in a sample displacement correction for this geometry.

PDFs were generated from the Si collected in a 0.3 mm and 1 mm capillary to determine the effect that capillary size and effective angular resolution have on the peak FWHM across *r*. The peak width is convoluted with an instrument broadening term in real space and can be described using equation (4) if the *Q*-dependent broadening is linear, where σ_0_ is the FWHM of a peak, δ is the Gaussian broadening term (*Q*_broad_) and σ_*q*_ is the FWHM of a peak convoluted with the broadening term (Thorpe *et al.*, 2002 ▸). The σ_*q*_ of the first peak at 2.35 Å was used to determine σ_0_ and establish the FWHM as a function of *r* from equation (4). Fig. S3(*b*) shows that the FWHM increases as *r* increases and is larger for the 1 mm capillary. At low *r* there is only a small difference in the peak widths (0.04 Å at *r* = 50 Å) between the capillary sizes and this difference increases due to peak broadening effects across *Q*,

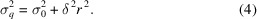

**Figure 1:**
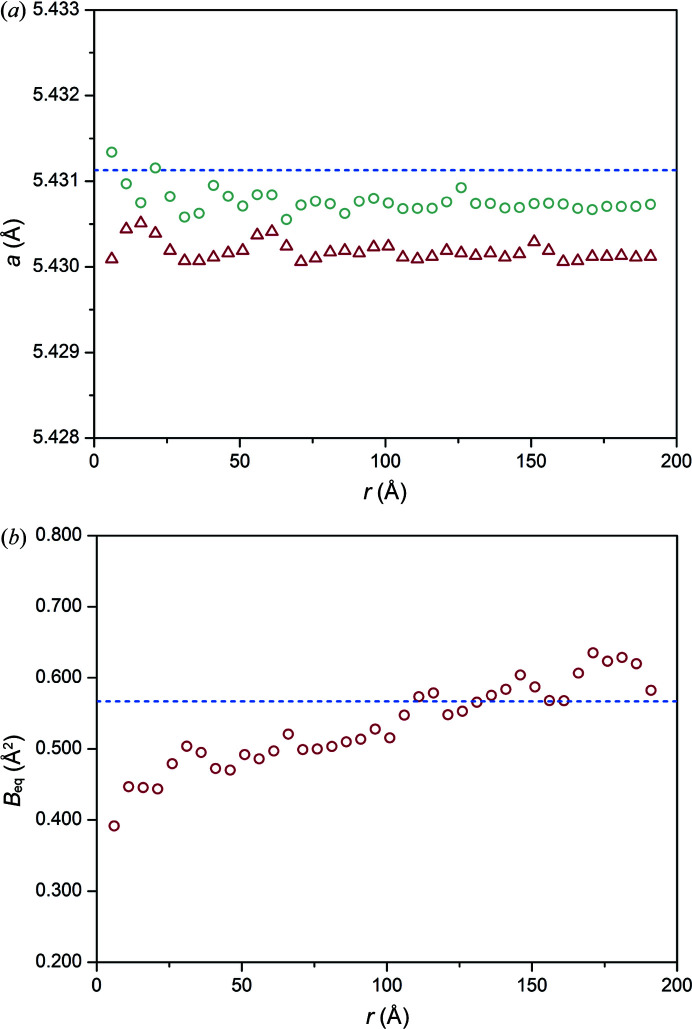
Data obtained from NIST Si 640d show (*a*) the PDF- and Bragg peak-derived lattice parameters are comparable and the PDF-derived value does not significantly change across *r*, where the circles are the lattice parameters corrected for peak shape and zero offset, and the triangles are the lattice parameter without correcting for peak shape and zero offset; and (*b*) the ADP increases across *r* due to peak broadening. The blue dashed lines indicate the lattice parameter and ADP obtained from Rietveld refinement.

In this work, the lattice parameters, atomic displacement parameters (ADPs) and scale factor were calculated in ‘box-car’ refinements using 10 Å windows and moving the centre of the ‘box’ by 5 Å. The dampening and broadening parameters were set to zero as this allowed us to determine how these parameters change across *r*. Lattice parameters were determined with and without correcting for instrumental effects on the peak profile and zero offset. In both, the lattice parameter was consistent across *r* [Fig. 1 ▸(*a*)]. The lattice parameters obtained by fitting the PDF without corrections were lower than the value of 5.4311 (1) Å from the Rietveld refinement, although they were comparable. Fig. S1 shows the fit to the NIST Si 640d standard in reciprocal space. The lattice parameter was 5.4302 (1) Å by refining between 1 Å and 100 Å, and at low (5 Å) and high (200 Å) *r* was ∼0.02% or 0.001 Å lower than the lattice parameter calculated from the Rietveld refinement of the Bragg peaks. This difference between the lattice parameter calculated from the PDF and Bragg peaks can be attributed to a zero offset and peak asymmetry in reciprocal space. The PDF was then generated and corrected for the peak profile described by fundamental parameters and zero offset using *TOPAS* (version 7; Coelho, 2020 ▸, 2021 ▸). The corrected lattice parameter was 5.4307 (1) Å by refining between 1 Å and 100 Å and at low (5 Å) and high (200 Å) *r* was <0.01% or <0.001 Å lower than the lattice parameter calculated from Rietveld refinement of the Bragg peaks. The Bragg peaks in the data from the PD beamline can have peak asymmetry that attributed to to axial divergence that can be modelled using a circles function via a fundamental parameters approach (Cheary & Coelho, 1992 ▸). In both real and reciprocal space, the peak position is determined from a delta function convoluted with line shape. The line shape has instrument and sample contributions. If an asymmetric line shape is not considered in the model then the intensity centre position can be different from the ‘ideal’ peak position obtained from the delta function only (Jeong *et al.*, 2005 ▸). Larger differences between the lattice parameter calculated from real and reciprocal space data can then be observed, as shown in this work. Asymmetric peak shapes can also cause *r*-dependent peak shifts, a reduction in the ability to fit peaks at high *r* (shown by a higher *R*_wp_) and changes to dampening behaviour (Olds *et al.*, 2018 ▸; Jeong *et al.*, 2005 ▸). The PDF-derived lattice parameter in this work shows good agreement with the Rietveld-derived lattice parameter once the data were corrected for peak profile and zero offset effects. Consequently, PDFs should be corrected for instrumental effects to minimize unwanted effects in the PDF.

There was an increase in the ADP across *r* showing an increase in peak width attributed to *r*-dependent peak-broadening [Fig. 1 ▸(*b*)]. A decrease in scale factor at high *r* may also indicate dampening of the peaks (Qiu *et al.*, 2004 ▸) [Fig. S4(*a*)]. The ADP was 0.567 (2) Å^2^ determined from Rietveld refinement and comparable to the 0.553 (1) Å^2^ from the PDF by refining the data between 1 Å and 100 Å [Fig. 1 ▸(*b*)]. Fig. S4(*b*) also shows the change in *R*_wp_ across *r*. Both dampening and broadening of the peaks due to the instrument can be accounted for using a standard and the values determined applied to samples during analysis.

### 3.2. Effect of *Q*-range

An energy of 21 keV is typically used for measurements at the PD beamline as flux dops off significantly above 22 keV. Ideally, all samples are measured at the highest available energy to increase the momentum transfer, *i.e.* to obtain a high *Q*_max_ and low *Q*_min_. The ability to resolve two peaks in a PDF is primarily determined by the *Q* range.

*Q*_min_ = 0.37 Å^−1^ (*d* ≃ 17 Å) can be reached if the data are measured from 2° 2θ at 21 keV. Angles lower than 2° 2θ cannot be accessed if also measuring to the higher angles needed for total scattering experiments, as scattering from our current beam stop is observed at higher angles (typically >60° 2θ). However, for reciprocal space measurements, data from 1° 2θ (*d* ≃ 33 Å) are routinely collected for materials with large unit cells by positioning the beam stop to reduce the small angle scattering and not cut the background intensity. The PD beamline is considering modifying the beam stop to reach lower angles while also avoiding backscatter. There is a trade-off between accessing low and high angles in the beamline setup with our current beam stop, so for total scattering experiments *Q*_min_ = 0.5 Å^−1^ should be used to generate the PDFs.

Measuring samples at an energy lower than 21 keV was considered in this work if samples contain elements that will have significant fluorescence when measured at 21 keV. The Mythen-II detector is sensitive to fluorescence, so the contribution of a fluorescence background to the measured data is minimized if the energy selected is below the absorption edge or greater than 6 keV above the absorption edge (Bergamaschi *et al.*, 2010 ▸). However, using a lower energy will obviously reduce the achievable *Q*_max_ and decrease the *Q*_max_ used, and hence cause an increase in peak-broadening, decrease in peak height and increase in the intensity of termination ripples (Qiu *et al.*, 2004 ▸).

**Figure 2:**
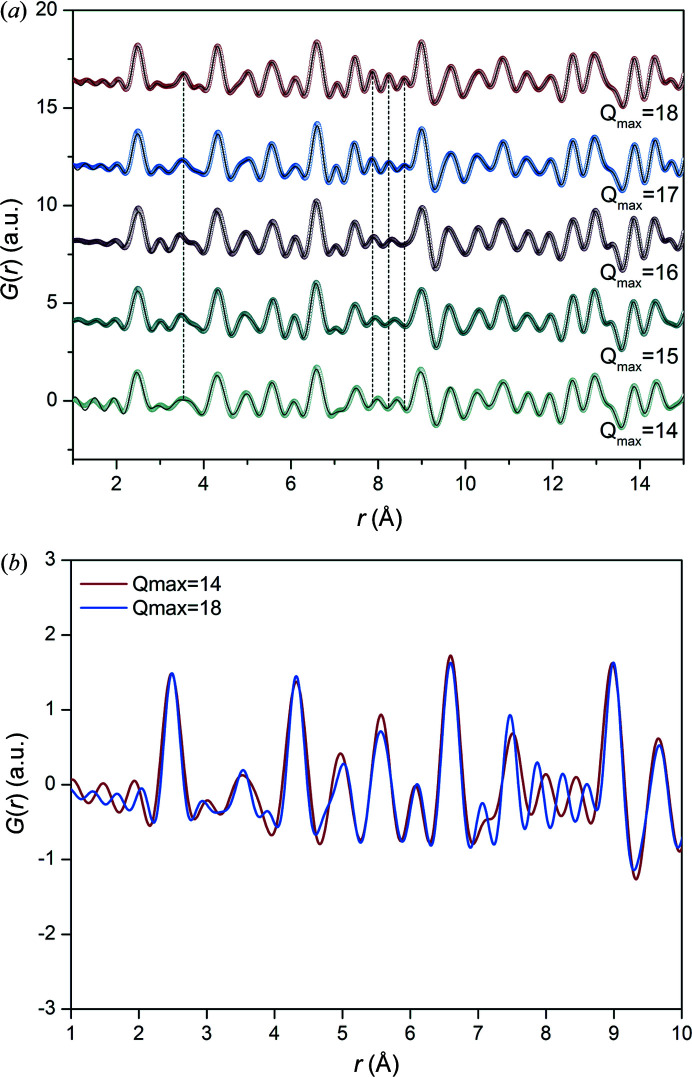
Data for an Ni sample show the (*a*) difference in resolution for *Q*_max_ = 14 Å^−1^ to 18 Å^−1^, and (*b*) loss of resolution observed for *Q*_max_ = 14 Å^−1^ compared with *Q*_max_ = 18 Å^−1^. In (*a*) the dotted plot is the measured data, and the line (black) shows the calculated model.

The extent to which the loss in real space resolution and variation in the calculated structural parameters increases was investigated for experiments where lower energies are considered. To investigate this, PDFs of an Ni powder sample were generated with *Q*_max_ = 14 Å^−1^, 15 Å^−1^, 16 Å^−1^, 17 Å^−1^ and 18 Å^−1^. The instrument parameters were also refined for each *Q*_max_. Fig. 2 ▸(*a*) shows that there is little to no observable difference between PDFs with *Q*_max_ = 17 Å^−1^ and 18 Å^−1^. There are three peaks between 7.7 Å and 8.7 Å in both of these PDFs, and the loss in resolution is obvious when *Q*_max_ ≤ 16 Å^−1^ as there are only two peaks visible in this region. The loss in resolution is also highlighted by the peak at 3.5 Å for *Q*_max_ = 14 Å^−1^ where the peak and termination ripples cannot be distinguished from each other. Resolution in real space can be estimated by δ*r* = 2π/*Q*_max_ (Petkov, 2012 ▸) so the resolution achieved using *Q*_max_ = 18 Å^−1^ is 0.35 Å, and for *Q*_max_ = 16 Å^−1^ it is 0.39 Å. This shows that only differences between correlation lengths that are >0.35 Å can be resolved at the PD beamline.

Changes in the lattice parameter and ADP were quantitatively evaluated by fitting the PDF data between *r* = 1 Å and *r* = 30 Å. The refined parameters are presented in Table S1. The lattice parameters for *Q*_max_ = 14–18 Å^−1^ are comparable and the ADPs increased when *Q*_max_ decreased. Peaks appear visibly broader at 2.5 Å and 4.3 Å for *Q*_max_ = 14 Å^−1^ compared with *Q*_max_ = 18 Å^−1^ [Fig. 2 ▸(*b*)]. Larger ADPs are obtained from broader peaks as they indicate atomic motion or static disorder. Correlations in the PDF broaden due to these effects as well as termination of the function at *Q*_max_. This is due to convolution of *G*(*r*) with a broadening function, sin(*Q*_max_Δ*r*)/Δ*r*.

A low *Q*_max_ resulted in the highest discrepancy between the ADPs of an experimental and a calculated PDF (Toby & Egami, 1992 ▸). Termination broadening can be reduced and is minimal if a high enough *Q*_max_ is used. Toby & Egami (1992 ▸) report that the errors in a PDF are minimal when *Q*_max_ > 30 Å^−1^. Qiu *et al.* (2004 ▸) showed from neutron PDF data of Pb that the peak intensity of the next-nearest neighbour at 3.48 Å does not increase further above *Q*_max_ = 37 Å^−1^ as the resolution is no longer measurement-limited. However, it is important to note that this is material-dependent. Consequently, the ADPs determined here are larger at lower *Q*_max_ due to peak-broadening from termination of the PDF.

Although PDFs can be generated with a lower *Q*_max_, the structural parameters for some materials may not be able to be determined owing to decreasing accuracy of the calculated parameters. Only qualitative data and relative changes between samples may be useable in this case. Users of the PD beamline will need to consider whether the achievable real space resolution is adequate to resolve the correlations of interest in their materials.

### 3.3. Absorption and the PDF

X-ray absorption effects are not generally an issue on higher-energy beamlines and those specifically designed for total scattering experiments. However, absorption of a sample needs to be considered when carrying out experiments on the PD beamline. For example, at 21 keV, CeO_2_ in a 0.3 mm capillary has μ*R* = 1.59, compared with μ*R* = 0.52 that can be achieved on other beamlines that reach 60 keV. Data quality is lower for highly absorbing samples in Debye–Scherrer geometry as the Bragg intensity is dependent on the diffraction angle and scattering may not be from the entire sample volume (Von Dreele & Rodriguez-Carvajal, 2008 ▸).

Any absorption contribution needs to be removed from the measured intensity in a total scattering experiment prior to Fourier transform. *S*(*Q*) needs a multiplicative correction applied to remove absorption effects that dampen the intensity at high *Q* (Peterson *et al.*, 2003 ▸). Using the *ad hoc* method of data correction in this work relies on the input of data without absorption effects (Juhás *et al.*, 2013 ▸). Although minor absorption can be corrected using standard data analysis programs, the significant peak intensity reduction observed in a highly absorbing sample often means meaningful results cannot be obtained.

Absorption can be minimized by employing a smaller internal diameter capillary or diluting the sample with a known material to reduce the quantity of highly absorbing material. An alternative method used to reduce μ*R* of highly absorbing samples by the XRD1 beamline (5.5–14 keV) at Laboratório Nacional de Luz Síncrotron involved fixing a thin layer of the material onto the outside of a capillary rather than filling the capillary (Carvalho *et al.*, 2017 ▸). Generally, diluting with an amorphous material such as amorphous SiO_2_ to obtain μ*R* < 1 is recommended as sample preparation is more straightforward and μ*R* can be decreased further than is achievable using a smaller internal diameter capillary. However, the addition of amorphous diluents will contribute to the PDF so the measured background should account for this by being an equivalent capillary filled with dilutant. The effect of absorption on the resultant PDF pattern was investigated using powdered Cu (undiluted) with μ*R* ≃ 2.27 (packing density = 5.28 g cm^−3^) and Cu (diluted), which was mixed with amorphous SiO_2_ to reduce the absorption so that μ*R* ≃ 0.9 (Cu + SiO_2_). A capillary filled with amorphous SiO_2_ was removed as the background.

**Figure 3:**
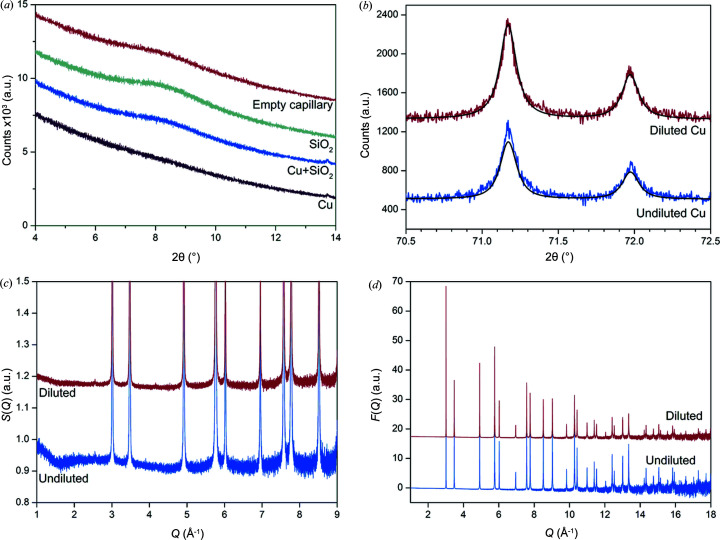
Error is introduced into the PDF if highly absorbing samples are not diluted. (*a*) Comparison in baseline between the empty borosilicate capillary, SiO_2_, diluted (Cu + SiO_2_) and undiluted Cu; (*b*) the diffracted intensities for undiluted Cu at high angles are stronger than what the structure model for Cu would predict; (*c*) *S*(*Q*); and (*d*) *F*(*Q*) showing the difference in background subtraction of Cu.

A Rietveld refinement was carried out on the diluted and undiluted Cu using a Pearson VII to describe the peak shape. The structural parameters are presented in Table S2. The refined ADP for the undiluted sample (0.296 Å^2^) was lower than that determined for the diluted Cu (0.517 Å^2^) when the ADPs were refined. However, when the ADP obtained for the diluted sample was used for the undiluted Cu and fixed [Fig. 3 ▸(*b*)], the structural model of the undiluted Cu predicted the high-θ reflections were lower intensity. The low-θ reflections in the undiluted Cu are lower in intensity due to absorption, so the model fits these while underestimating the intensity of the high-angle peaks. Relative absorption decreases as θ increases as the high-angle reflections can diffract from the surface and reduce absorption (Cullity, 1978 ▸). These results show that absorption is greater for low-angle reflections as they have a longer pathlength through the sample.

Figs. 3 ▸(*c*) and 3 ▸(*d*) show there are additional artefacts in *S*(*Q*) and *F*(*Q*) due to poor background subtraction when the absorption contribution is not removed. Peterson *et al.* (2003 ▸) showed that a sine wave can be introduced into the *S*(*Q*) from a poorly corrected background. The artefacts manifest as a curve in the baseline of these functions and propagate into the PDF. The increased noise is also higher in the undiluted sample as shown in Fig. 3 ▸(*d*). This is likely from lower counting statistics as more photons are absorbed by the sample so fewer are reaching the detector. The scattering from the empty capillary shows the characteristic broad amorphous peak at 7–9° 2θ [Fig. 3 ▸(*a*)] like the scattering of the diluted Cu. The undiluted Cu pattern does not exhibit this broad capillary scattering peak and the model does not appear to fit the data well at low *r*. This indicates an increase in the error of the low-*r* peak intensities. Figs. S5(*a*) and S5(*b*) show that the fit to the undiluted Cu PDF is poorer compared with the Cu diluted with SiO_2_ as demonstrated by difference plot and higher *R*_wp_. The difference in fit quality also has a clear effect on the calculated ADP as shown in Table S3. Users of the PD beamline will need to dilute highly absorbing samples to minimize absorption and improve the quality of their PDF.

### 3.4. Counting time duration

**Figure 4:**
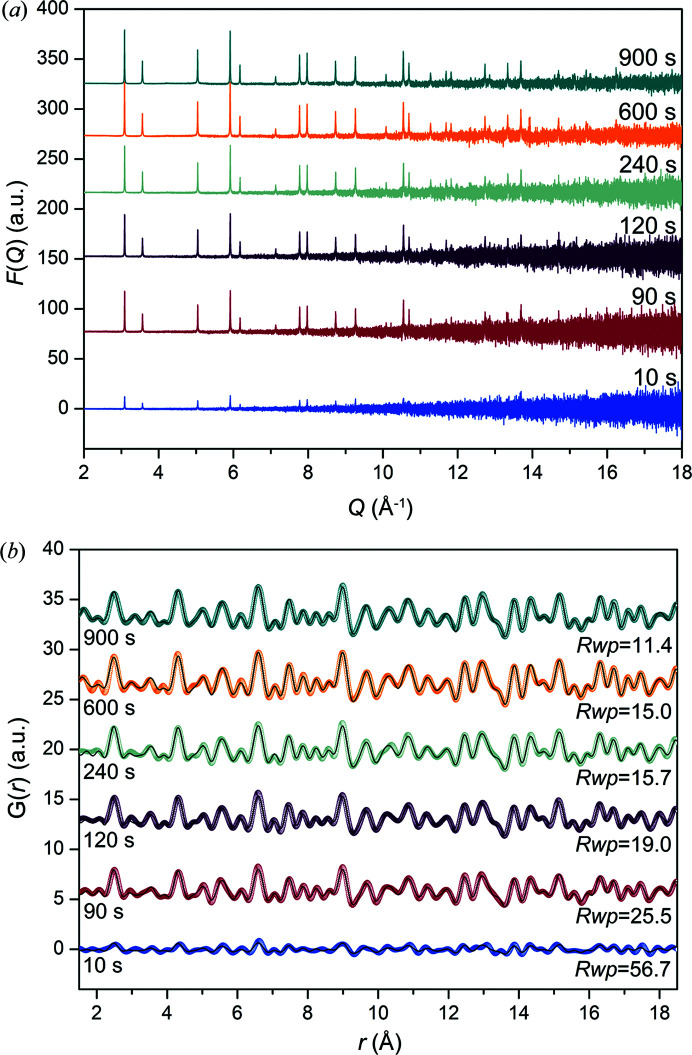
Data for the Ni particles showing (*a*) noise in *F*(*Q*) at higher *Q* decreases as the counting time increases, and (*b*) how the increase in statistical error manifests in *G*(*r*). For (*b*), the dotted plot is the measured data and the line (black) shows the refined model.

Data for the Ni particles were collected for 10–900 s to understand the effect of counting time on acceptable counting duration for stable samples. Note that counting time is highly sample-dependent but is generally longer than is required for reciprocal space measurements. It is well known that a longer measurement time will lead to better counting statistics, so the optimum counting time needs to be balanced with an acceptable counting time duration. This is also important at large-scale facilities where the total beam time is limited. *F*(*Q*) shows that the noise at higher *Q* decreases as counting time increases from a reduction in statistical error [Fig. 4 ▸(*a*)], as expected. The resultant PDFs in Fig. 4 ▸(*b*) show that the intensity of the ripples relative to the peak intensity decreased for the PDFs obtained with the higher counting time of 900 s compared with 10 s. Most noticeable is the lower-quality PDF obtained when collecting for only 10 s. The peaks at higher *r* cannot be differentiated from the ripples in the background and these prominent ripples contribute to the correlation peak shape. The first correlation at 2.5 Å is asymmetric due to the contribution of large ripples to the peak profile. Interpreting a PDF peak profile with poor counting statistics is problematic as these peaks are convoluted with a sine function that cannot always be modelled well and this will affect the refined parameter values. Consequently, a low counting time affects the quality of the PDF and the calculated structural data.

The lattice parameters determined from modelling the PDFs were comparable for all counting times (Table S4). However, the PDF collected for 900 s has the lowest *R*_wp_. These results also exemplify that counting times are sample dependent. *R*_wp_ was lower for Ni (Table S1) powder than for the Ni particles when all were collected for 240 s. The total number of Ni atoms per sample volume interacting with the beam is lower for the Ni particles (20 wt%) due to the presence of carbon. Hence counting times for supported nanoparticle samples can be longer if the scattering from the particles is weak from dilution by the support, the particles are a few nanometres in diameter and they are dispersed. Longer counting times that ensure good counting statistics above 90° 2θ may also result in backscatter contributing to the peak intensities in reciprocal space. It is important to ensure that the collected data are free from backscattering as this contribution can undesirably contribute to the PDF, hence contaminating the data. A compromise is needed between the optimum acquisition time and data quality, so it is suggested that an acquisition time of 600 s is acceptable for similar samples.

### 3.5. Nanoparticle case study

Bimetallic Pt-based nanoparticles have applications as electrocatalysts for formic acid and ethanol electro-oxidation and oxygen reduction. Nanoparticles such as NiPt are reported to have superior performance compared with monometallic nanoparticles due to synergistic effects between their elements as well as typically exhibiting a reduction in surface poisoning (Zhang *et al.*, 2018 ▸). The PDFs of a series of metal particles, Ni, NiPt, dealloyed NiPt and Pt were obtained to establish the data quality for nanomaterials at the PD beamline. The PDF crystallite sizes were compared with those obtained from TEM and the first three correlation lengths were compared with those calculated using XAS.

#### 3.5.1. Crystallite size distribution and morphology

**Figure 5:**
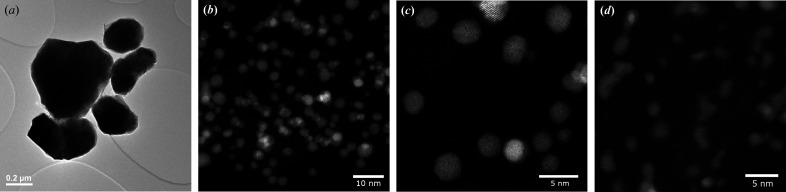
(*a*) Ni consists of large crystallites as shown in the BF-TEM compared with (*b*) NiPt, (*c*) dealloyed NiPt and (*d*) Pt nanoparticles from the STEM-HAADF images.

The crystallite size distribution and morphology were obtained for the NiPt, dealloyed NiPt and Pt nanoparticles using STEM. The HAADF signal in STEM mode is proportional to the atomic number so measuring the crystallite size is facilitated using STEM. Both NiPt and dealloyed NiPt possess round and facetted single crystallites as shown in Figs. 5 ▸(*b*) and 5 ▸(*c*), whereas the morphology of Pt appears more variable [Fig. 5 ▸(*d*)]. Images of Ni were obtained using BF-TEM and show that the particles are significantly larger than the NiPt, dealloyed NiPt and Pt nanoparticles [Fig. 5 ▸(*a*)]. These differences in crystallite size are reflected in the reciprocal and real space diffraction data. Fig. 8 shows that the Ni powder diffraction pattern has sharp Bragg peaks while the nanoparticles have broad peaks. The NiPt and dealloyed NiPt nanoparticles were also slightly larger than Pt as shown in Table 1 ▸. The particle diameter histograms are presented in Fig. S6, and additional STEM images are presented in Fig. S7.

#### 3.5.2. Local environment from XANES and EXAFS

**Figure 6:**
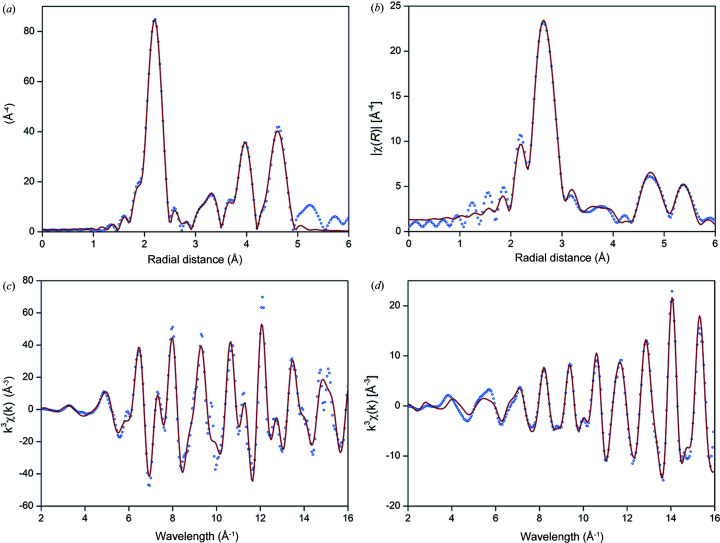
Radial distances determined using EXAFS for (*a*) Ni and (*b*) Pt, and the corresponding *k*-plots for (*c*) Ni and (*d*) Pt show good agreement with the PDF-determined correlation lengths. The dotted (blue) spectra are the measured data and the line (red) spectra is the fitted model.

A comparison with the structural parameters determined from PDF was established for the supported particles. The EXAFS data from Ni and Pt were fit using the predicted scattering of the neat face-centred cubic structures. The plotted results of the fit are given in Fig. 6 ▸, which show good agreement out to 5 Å and the third shell of atomic distances. The determined radial distances between the absorbing atom and the indicated shell are given in Table 2 ▸. EXAFS fit parameters of the Pt and Ni nanoparticle analysis are presented in Table S5. A comparison between the EXAFS radial distances and the PDF correlation length is discussed in Section 3.5.3.

**Figure 7:**
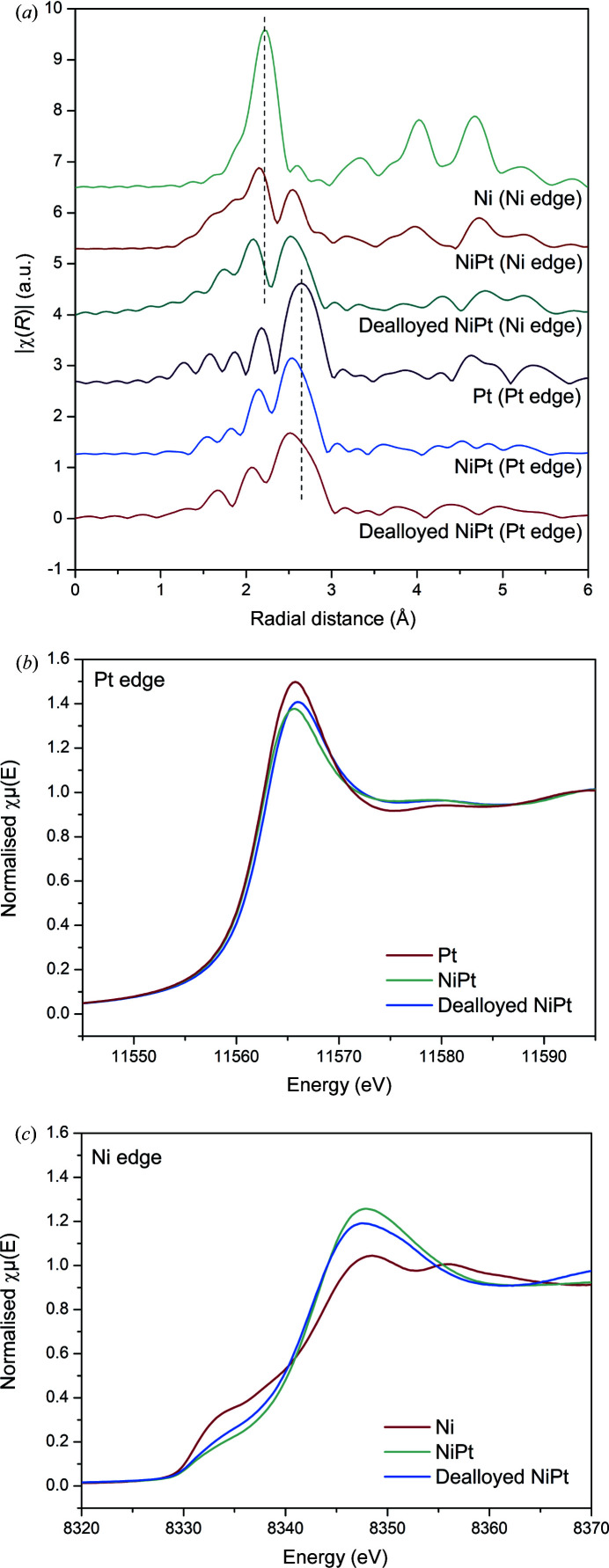
(*a*) EXAFS Ni- and Pt-edge comparison of the NiPt and dealloyed NiPt nanoparticles, and XANES plots of (*b*) Pt and (*c*) Ni.

The Ni phase and Pt phase can be probed by examining their respective edges. The PtNi nanoparticles were analysed at both edges and compared with the Ni and Pt nanoparticles. The spectra in Fourier transform (*R*) space are presented in Fig. 7 ▸(*a*) and the radial distance functions are presented in Fig. S8. It can be qualitatively observed that the Pt phase in the NiPt and dealloyed NiPt nanoparticles exists predominately as the neat metal, closely resembling the spectra for Pt. Both NiPt and dealloyed NiPt can be fit reasonably well with only scattering from Pt–Pt and Pt–O in the first shell without significant contribution from Ni. This would suggest that the nanoparticles contain a relatively pure Pt phase. This is further corroborated with a XANES analysis, in which the absorption edge is identical between the Pt nanoparticles and the NiPt and dealloyed NiPt [Fig. 7 ▸(*b*)]. In contrast, the Ni phase of the NiPt and dealloyed NiPt differ significantly from the pure nanoparticles and cannot be fit with only Ni–Ni and Ni–O scattering paths. The XANES analysis likewise shows a significant decrease in the pre-edge feature present in the Ni nanoparticles, behaviour which is associated with NiPt alloy formation [Fig. 7 ▸(*c*)]. This suggests that the Ni phase is not pure and has Pt–Ni alloy characteristics (Chen *et al.*, 2016 ▸).

#### 3.5.3. Correlation lengths from PDFs

**Figure 8:**
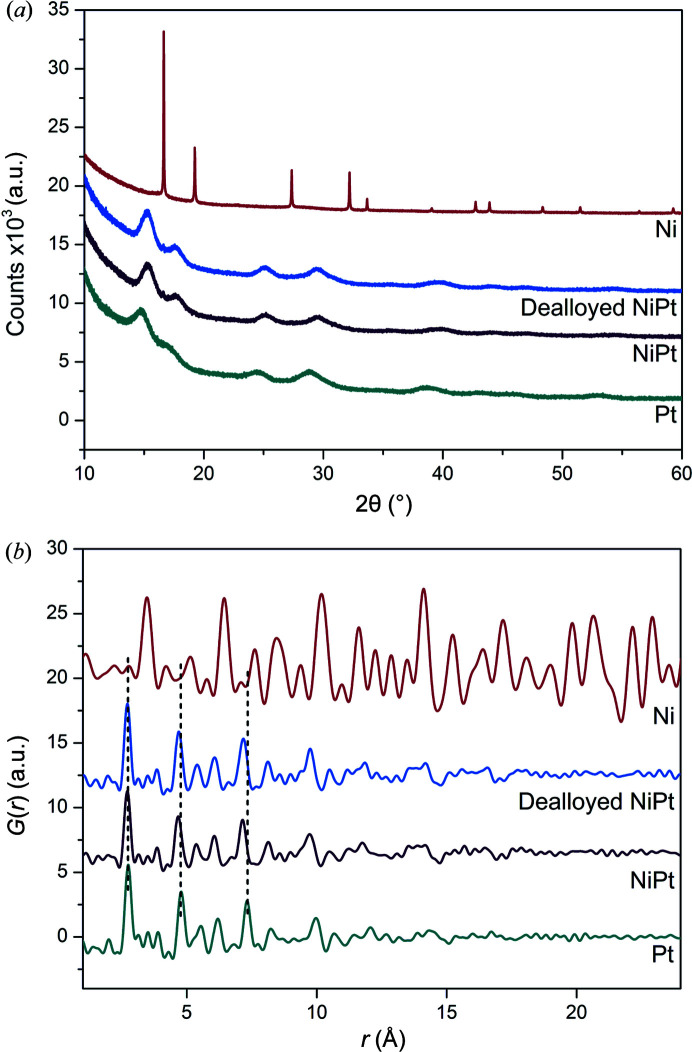
(*a*) Diffraction patterns and (*b*) PDFs of the Ni, NiPt, dealloyed NiPt and Pt nanoparticles.

Nanometre-sized crystallites have broad peaks as the diffracted intensity is spread further from the reciprocal lattice points. To minimize line broadening, crystallites are recommended to be 1–5 µm to maintain a good power average (McCusker *et al.*, 1999 ▸). Fig. 8 ▸(*a*) shows that only the Ni possesses relatively narrow Bragg peaks whereas the NiPt, dealloyed NiPt and Pt have broader peaks due to the size of the crystallites.

The PDFs of Ni and NiPt, dealloyed NiPt, and Pt nanoparticles are shown in Fig. 8 ▸(*b*). The larger crystallite size and long-range atomic order of Ni are reflected in the PDF as correlations do not attenuate like that observed for NiPt, dealloyed NiPt and Pt. The correlations for the nanoparticles dampen by ∼15 Å due to their finite crystallite size. The structures of all the nanoparticles from the PDFs were refined using the space group *Fm* 3 *m* (No. 225) and the occupancy in the 4*a* sites were split in the NiPt and dealloyed NiPt models, *i.e.* the occupancy for Ni was 0.5 and Pt was 0.5. The ADP of each Ni and Pt site in the NiPt and dealloyed NiPt model were constrained. All nanoparticles were corrected for finite crystallite size using a numerical spherical shape function (Usher *et al.*, 2018 ▸). Fits to the PDFs are shown in Fig. S9 and structural details in Table S6. Microstrain, structural defects and effects from the surface atom geometry which are often observed in nanomaterials may be present in these samples (Bertolotti *et al.*, 2018 ▸). There was no improvement to the fit when octahedral- and tetrahedron-shaped functions were tested and the TEM data show that the crystallites for NiPt and dealloyed NiPt were round and facetted. The size of the Pt nanoparticle sample was 1.9 nm, which corresponds well with the average particle size of 2.0 nm from TEM (Table 3 ▸). The values obtained from the PDF for PtNi (2.2 nm) and dealloyed PtNi (2.4 nm) are lower than the 3.1 nm obtained for both samples using TEM. A limitation of TEM in the particle-size distribution is that the number of particles sampled is orders of magnitude lower than the number that are sampled using X-ray diffraction. Particle sizes are obtained by measuring the diameter from a 1D image so the length along other axes is absent and may be a different length if the particle shape is irregular. However, accurate particle sizes from modelling diffraction data are also compromised if the fit quality is poor. A correct model and reasonable starting parameters are needed for least-squares minimization refinements from existing knowledge of the sample. The difference between the particle sizes from the PDF and TEM observed in this work are proposed to be caused by the difference between how the particle size is determined using both techniques.

The first three correlation lengths were determined from the refined Ni and Pt structural models. For Ni these were 2.48 Å, 3.54 Å and 4.31 Å, and for Pt they were 2.76 Å, 3.91 Å and 4.78 Å. Both the Ni and Pt correlation lengths agree well with the values obtained using EXAFS. Correlations for the NiPt and dealloyed NiPt nanoparticles are at larger *r* than the corresponding peaks for Ni, and smaller *r* than the corresponding peaks for Pt. This is due to the combination of the two elements with different atomic sizes in the samples. The correlations at 3.83 Å and 3.84 Å for the NiPt and dealloyed NiPt are at positions closer to those of the Pt (3.91 Å) rather than Ni (3.54 Å). This implies that the structure is closer to Pt, rather than Ni, and both NiPt and dealloyed NiPt are not a 50:50 random alloy. This conclusion is corroborated by EXAFS. We conclude that accurate correlation lengths can be determined from the total scattering data measured on the PD beamline.

## 4. Considerations

Total scattering data can be obtained on the PD beamline, although there are constraints to the capabilities offered. These include:

(i) Measurements require stable samples and longer acquisition times. Collection times on the PD beamline can be longer than at dedicated higher flux X-ray PDF beamlines. Data should be collected over 124° 2θ using the Mythen-II positioned at four different starting angles. It takes ∼2 min to physically move from 2.5° 2θ (position 2) to 51.5° 2θ (position 3, see Section 2.1) as the diffractometer moves the Mythen-II at 0.4° per second. This excludes the time it takes to measure the data. In the nanoparticle samples reported here, the total measurement time is ∼18 min (240 s per position for four positions plus 124 s to move the detector). In comparison, I15-1 at Diamond Light Source has *in situ* battery sample environments than can collect PDF data in 2 min (Diaz-Lopez *et al.*, 2020 ▸). This makes non-ambient/*in situ* PDF measurements at the PD beamline more challenging as the conditions must remain stable throughout the whole data collection.

(ii) Dilution is essential for highly absorbing samples. Artefacts will be observed in PDFs of highly absorbing samples that are not diluted. This work shows that there are significant effects on the resultant PDF for highly absorbing samples, with the most noticeable effect on the calculated ADPs. Absorption causes a reduction in low-angle Bragg peak intensities and inaccuracies in background subtraction. It is essential that highly absorbing samples are always diluted so that μ*R* < 1. Users at the PD beamline should calculate μ*R* of their samples at 21 keV if they are planning a total scattering experiment and dilute if necessary.

(iii) Correlation differences >0.35 Å can be resolved. Correlation lengths that differ by >0.35 Å can be resolved due to the maximum momentum transfer of the PD beamline. Users need to consider whether the real space resolution offered by the PD beamline at 21 keV and an angular range of 124° 2θ is appropriate for their experiment.

## 5. Summary

In this work we explored the capabilities of carrying out total scattering experiments on the PD beamline, including investigations into the maximum resolution (*Q*_max_) absorption effects and counting time. The maximum momentum transfer is 19 Å^−1^ if the data are collected at 21 keV. Systems typically challenging to analyse using the Bragg peaks can be measured on the PD beamline via total scattering experiments. However, the PDF capability of the beamline can be further developed. Future upgrades planned for the PD beamline include a Mythen-III detector that will reduce data collection time and improve data quality. It includes an increase in angular range (150° 2θ) that will achieve a higher momentum transfer of 21 Å^−1^ if the data are collected at 22 keV for PDF studies. The Mythen-II requires two patterns to be collected, which are spliced together and normalized to remove gaps in the data from physical gaps between the modules. The measurement time will be halved using the Mythen-III as data without gaps between the modules will be obtained in a single pattern. These results detail the total scattering capability offered and should be used as a guide for those considering and designing total scattering experiments at the PD beamline.

## Supplementary Material

Figures S1 to S9 and Tables S1 to S6. DOI: 10.1107/S1600577522011614/gy5037sup1.pdf

## Figures and Tables

**Table 1:** Crystallite size of the nanoparticles determined from STEM

Sample	Size (nm)	Number of particles
NiPt	3.1 (1)	532
Dealloyed NiPt	3.1 (1)	310
Pt	2.0 (1)	452

**Table 2:** EXAFS radial distances of Ni and Pt Here *R* is the radial distance and σ^2^ is the Debye–Waller factor.

	Ni–Ni		Pt–Pt	
	*R* (Å)	σ^2^ (Å^2^)	*R* (Å)	σ^2^ (Å^2^)
First shell	2.48 ± 0.01	0.002	2.77 ± 0.01	0.003
Second shell	3.51 ± 0.01	0.003	3.92 ± 0.01	0.004
Third shell	4.32 ± 0.01	0.004	4.80 ± 0.01	0.007

**Table 3:** Crystallite size of the nanoparticles determined from PDF

	PtNi	Dealloyed PtNi	Pt
Sphere length (nm)	2.2 (1)	2.4 (1)	1.9 (1)
