# Supplementary material for: Total scattering measurements at the Australian Synchrotron Powder Diffraction beamline: capabilities and limitations
Source: J Synchrotron Radiat. 2023 Jan 13;30(Pt 2):327–39. doi: 10.1107/S1600577522011614 (PMC10000805; doi:10.1107/S1600577522011614)
Supplement: Supplementary file 1 [file s-30-00327-sup1.pdf]

JOURNAL OF SYNCHROTRON RADIATION

Volume 30 (2023)

Supporting information for article:

Total scattering measurements at the Australian Synchrotron Powder Diffraction beamline: Capabilities and limitations

Anita M. D'Angelo, Helen E. A. Brand, Valerie D. Mitchell, Jessica L. Hamilton, Daniel Oldfield, Amelia C. Y. Liu and Qinfen Gu

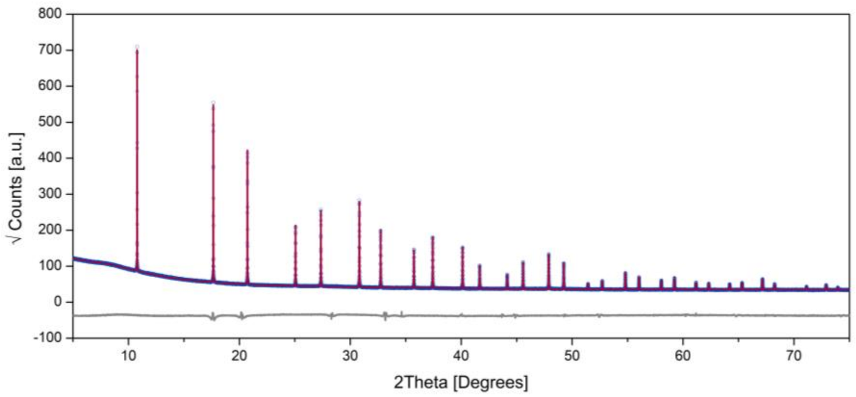

Figure S1 Fit to the NIST Si 640d data, where dotted (blue) is the measured data, the line (red) shows the calculated model and line below (grey) shows the difference plot.

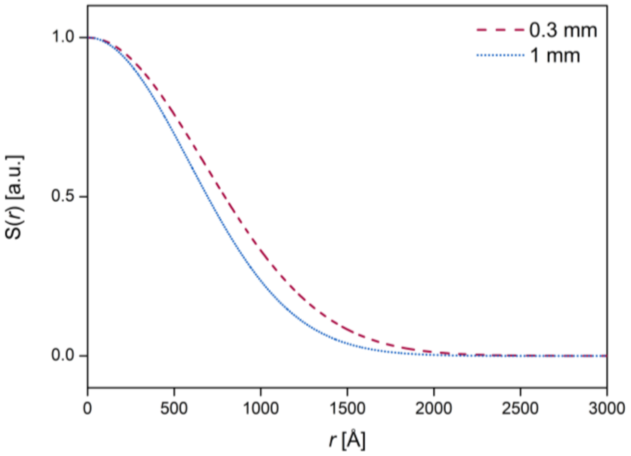

Figure S2 The *r* dependant scale factor determined for NIST Si 640d collected in a 0.3 mm (Q_{damp} = 0.0035) and 1.0 mm (Q_{damp} = 0.0040) capillary using Equation 3.

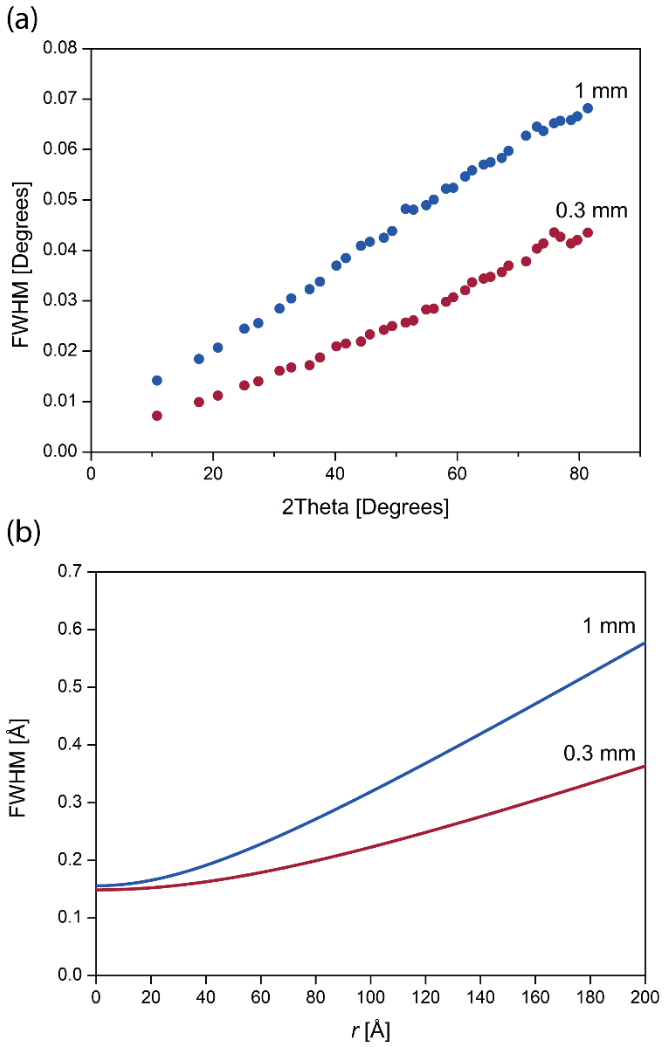

Figure S3 (a) Effective angular resolution in reciprocal space determined from NIST Si 640d, and (b) FWHM of PDF peaks as a function of *r* determined from a 0.3 mm (Q_{broad} = 0.0017) and 1.0 mm (Q_{broad} = 0.0028) capillary.

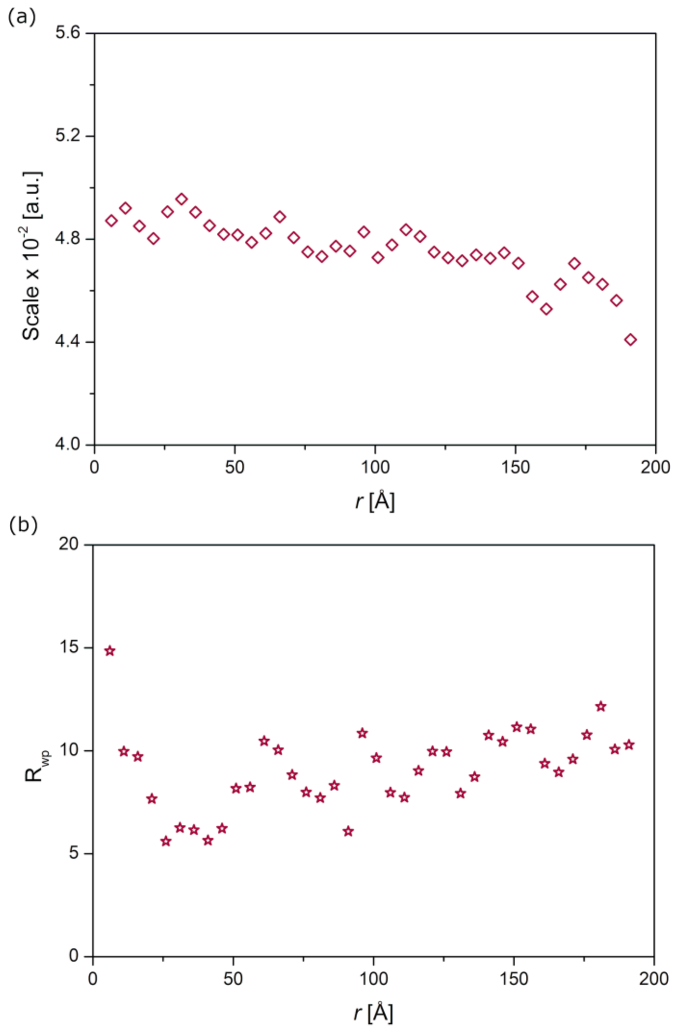

Figure S4 Data obtained from a NIST Si 640d shows the (a) scale and (b) R_{wp} change across r.

Table S1 Structural data from modelling the Ni PDFs generated at Q_{max} = 14 to 18 $Å^{-1}$ between 1–30 Å.

Q_{max}	**18**	**17**	**16**	**15**	**14**
R_{wp}	5.06	5.33	6.76	6.00	8.57
Delta2	1.84(1)	1.87(1)	1.91(2)	1.93(2)	1.67(2)
a (Å)	3.5251(1)	3.5250(1)	3.5250(1)	3.5251(1)	3.5252(1)
B_{iso} ($Å^2$)	0.333(1)	0.333(2)	0.338(3)	0.338(3)	0.340(4)

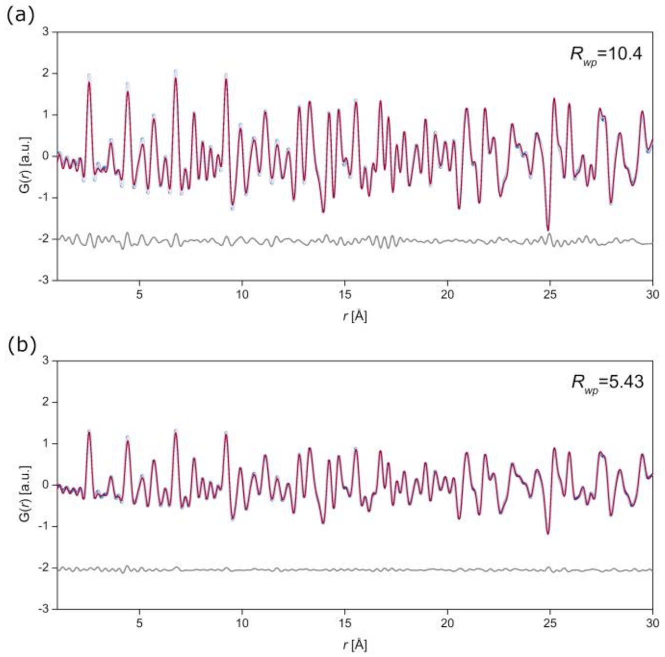

Figure S5 Absorption needs to be considered as the (a) fit to the data was poorer when Cu (with empty capillary background) was not diluted, compared to (b) the diluting Cu with SiO_2 (with SiO_2 filled capillary as background). The dotted (blue) plot is the measured data, the line (red) shows the calculated model and line below (grey) shows the difference plot.

Table S2 Fit parameters of diluted and undiluted Cu obtained via Rietveld refinement.

	**Undiluted Cu**	**Diluted Cu (Cu+SiO_2)**
R_{wp}	6.76	7.45
a (Å)	3.6151(1)	3.6152(1)
B_{iso} (Å^2)	0.296(6)	0.517(5)

Table S3 Fit parameters of diluted and undiluted Cu obtained from fitting the PDF.

	**Undiluted Cu**	**Diluted Cu (Cu+SiO_2)**
Background	Empty capillary	SiO_2 filled capillary
R_{wp}	10.4	5.43
Delta2	2.88(3)	3.26(2)
a (Å)	3.6146(1)	3.6147(1)
B_{iso} ($Å^2$)	0.397(4)	0.504(2)

Table S4 Fit parameters of Ni nanoparticle counting time comparison.

				**Ni**		
Counting time (s)	10	90	120	240	600	900
Numerical shape function				Sphere		
Background				Carbon filled capillary		
R_{wp}	56.7	25.5	19.0	15.7	15.0	11.4
a (Å)	3.524(1)	3.525(1)	3.523(1)	3.524(1)	3.525(1)	3.526(1)
B_{iso} ($Å^2$)	0.42(2)	0.36(1)	0.37(1)	0.37(1)	0.35(1)	0.39(1)

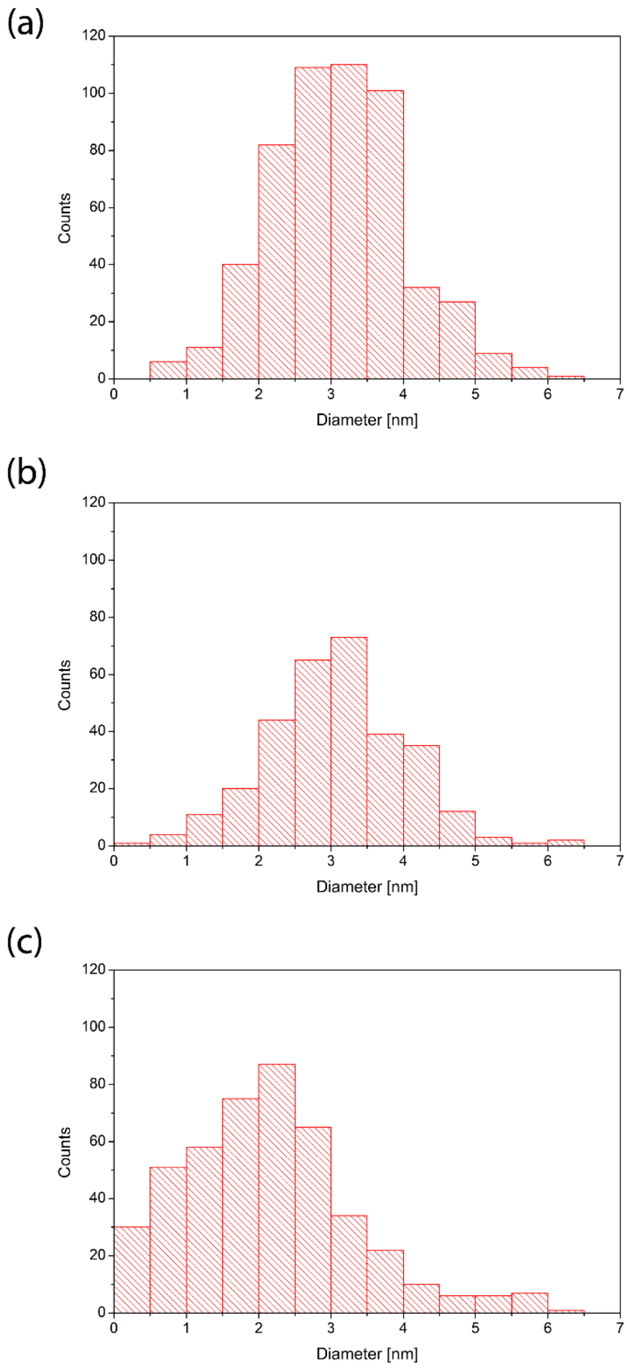

Figure S6 Particle diameter histogram from TEM for (a) NiPt, (b) dealloyed NiPt and (c) Pt.

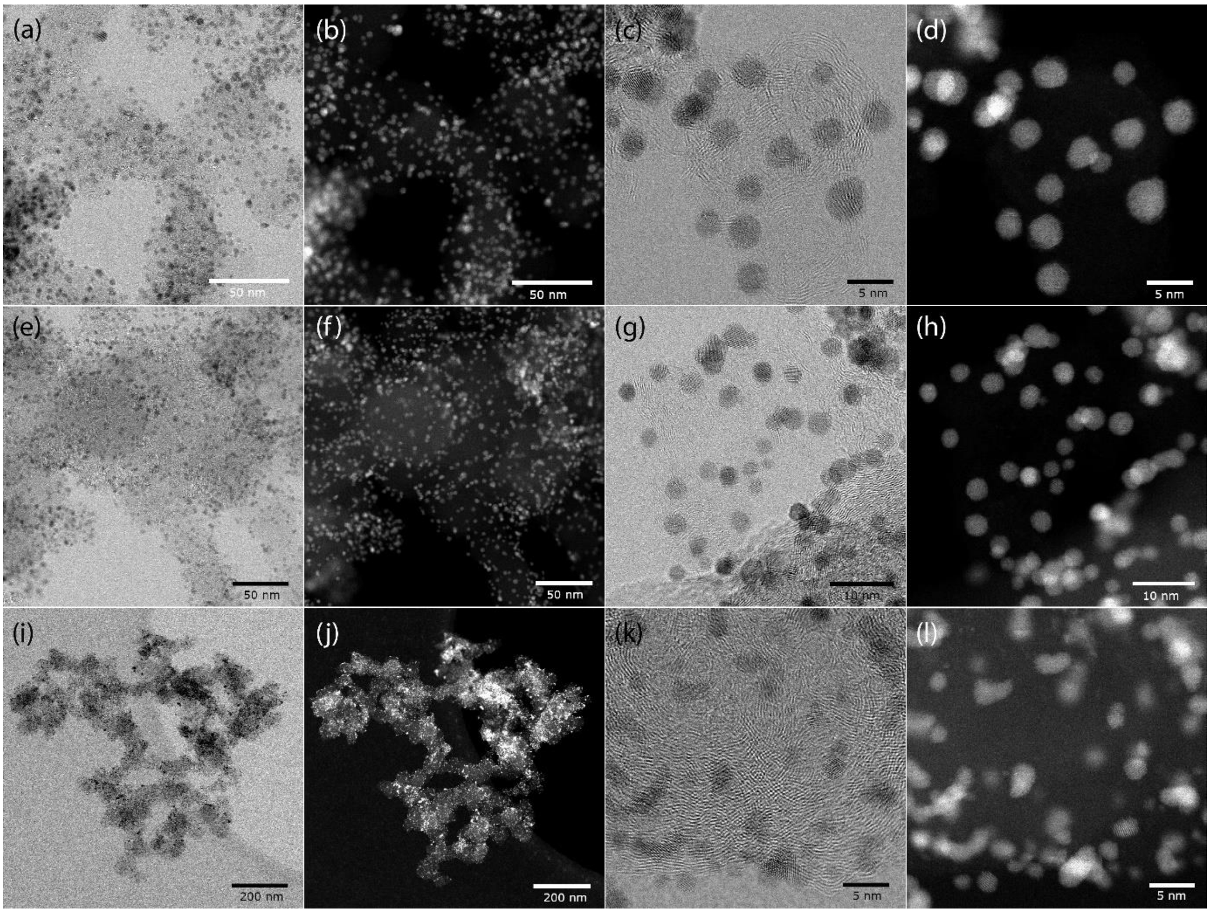

Figure S7 (a) Corresponding bright field and HAADF images of (a-d) NiPt, (e-h) dealloyed NiPt, and (i-l) Pt.

Table S5 EXAFS fit parameters of the Pt and Ni nanoparticle analysis.

	**Pt**	**Ni**
Independent points	27	37
Number of variables	14	16
Chi-square	446.2	616.0
Reduced chi-square	33	30
R-factor	0.004	0.005
k-weight	3	3

Table S6 Fit parameters of the PtNi, dealloyed PtNi and Pt nanoparticles from 1–15 Å. Data for Ni collected for 240 seconds is reported in Table S4.

	**PtNi**	**Dealloyed PtNi**	**Pt**
Numerical shape function		Sphere	
Background		Carbon filled capillary	
R_{wp}	24.3	22.0	32.1
Delta2	4.37(1)	4.73(6)	3.62(12)
a (Å)	3.827(1)	3.835(1)	3.911(1)
B_{iso} Ni, Pt (Å^2)	0.88(1)	0.97(1)	-
B_{iso} Pt (Å^2)	-	-	0.89(3)

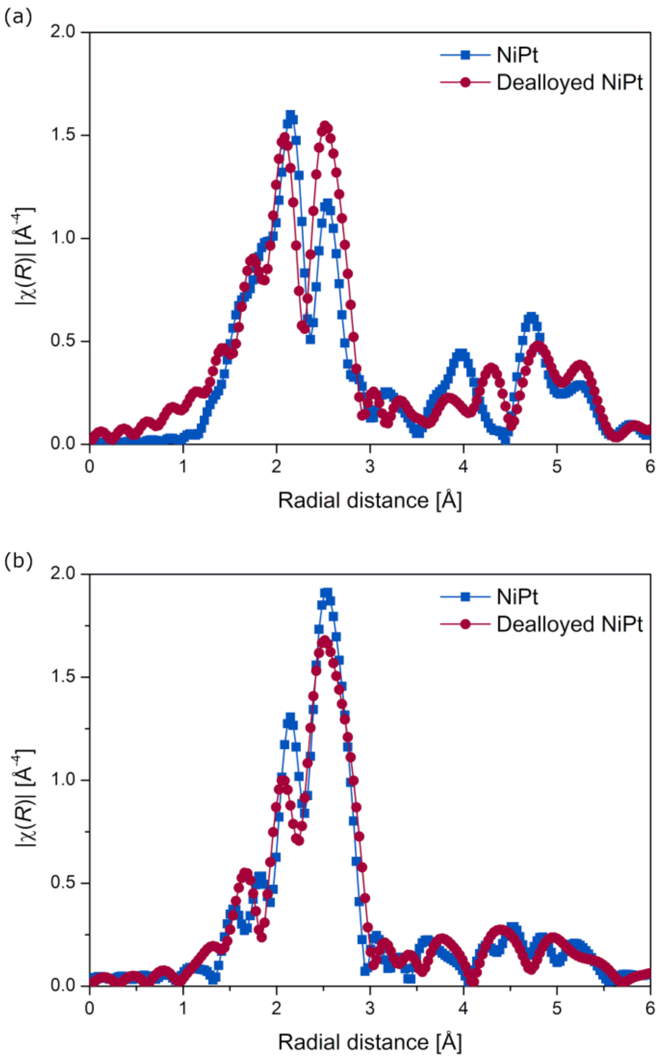

Figure S8 Comparison of the EXAFS radial distances for the (a) NiPt and (b) dealloyed NiPt nanoparticles.

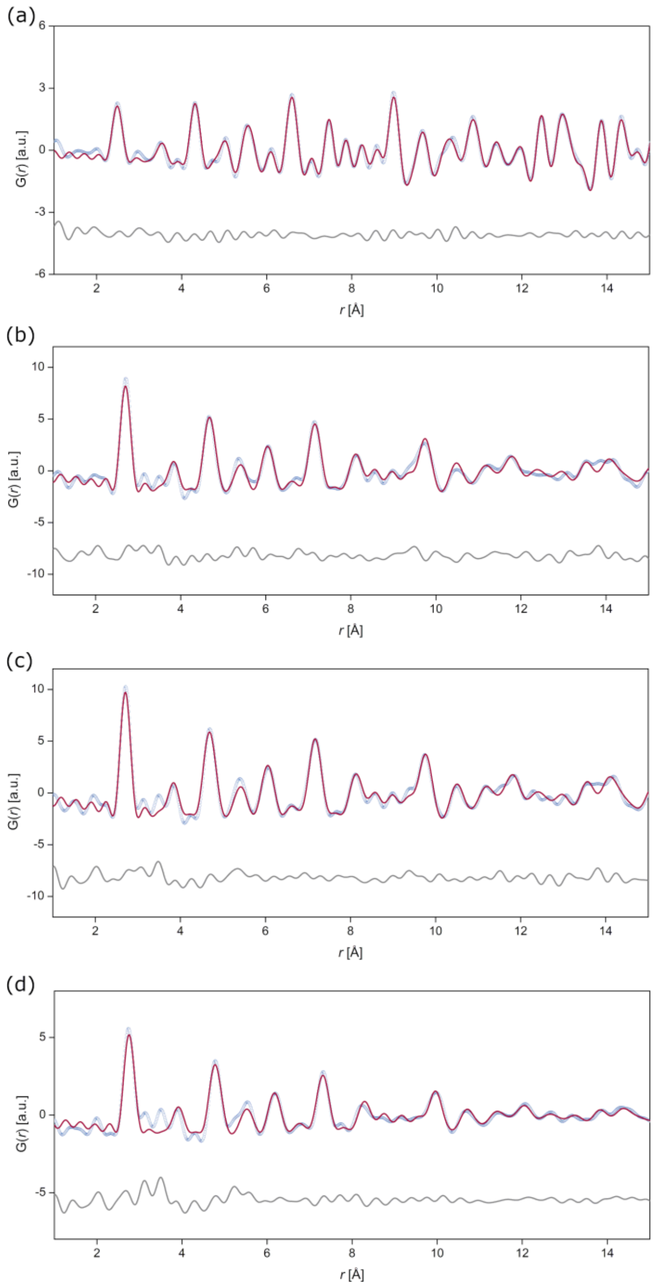

Figure S9 PDF fits of (a) Ni particles, (b) NiPt, (c) dealloyed NiPt, and (c) Pt nanoparticles. The dotted (blue) plot is the measured data, the line (red) shows the calculated model and line below (grey) shows the difference plot.
